# Review of Heat Transfer Analysis in Different Cavity Geometries with and without Nanofluids

**DOI:** 10.3390/nano12142481

**Published:** 2022-07-19

**Authors:** Farhan Lafta Rashid, Ahmed Kadhim Hussein, Emad Hasani Malekshah, Aissa Abderrahmane, Kamel Guedri, Obai Younis

**Affiliations:** 1 Petroleum Engineering Department, College of Engineering, University of Kerbala, Karbala 56001, Iraq; farhan.lefta@uokerbala.edu.iq; 2 Mechanical Engineering Department, College of Engineering, University of Babylon, Babylon City 51002, Iraq; ahmedkadhim7474@gmail.com; 3 College of Engineering, University of Warith Al-Anbiyaa, Karbala 56001, Iraq; 4 Department of Power Engineering and Turbomachinery, Silesian University of Technology, 44-100 Gliwice, Poland; emad.hasani@polsl.pl; 5 Laboratoire de Physique Quantique de la Matière et Modélisation Mathématique (LPQ3M), University of Mascara, Mascara 29000, Algeria; a.aissa@univ-mascara.dz; 6 Mechanical Engineering Department, College of Engineering and Islamic Architecture, Umm Al-Qura University, Makkah 21955, Saudi Arabia; kmguedri@uqu.edu.sa; 7 Department of Mechanical Engineering, College of Engineering at Wadi Addwaser, Prince Sattam Bin Abdulaziz University, Wadi Addwaser 11991, Saudi Arabia; 8 Department of Mechanical Engineering, Faculty of Engineering, University of Khartoum, Khartoum 11111, Sudan

**Keywords:** heat transmission augmentation, nanofluids, cavity, natural convection, forced convection, mixed convection

## Abstract

Many strategies have been attempted for accomplishing the needed changes in the heat-transfer rate in closed cavities in recent years. Some strategies used include the addition of flexible or hard partitions to the cavities (to split them into various pieces), thickening the borders, providing fins to the cavities, or altering the forms or cavity angles. Each of these methods may be used to increase or decrease heat transmission. Many computational and experimental investigations of heat transport in various cavity shapes have been conducted. The majority of studies focused on improving the thermal efficiency of heat transmission in various cavity containers. This paper introduced a review of experimental, numerical, and analytical studies related to heat transfer analyses in different geometries, such as circular, cylindrical, hexagonal, and rectangular cavities. Results of the evaluated studies indicate that the fin design increased heat transmission and sped up the melting time of the PCM; the optimal wind incidence angle for the maximum loss of combined convective heat depends on the tilt angle of the cavity and wind speed. The Nusselt number graphs behave differently when decreasing the Richardson number. Comparatively, the natural heat transfer process dominates at Ri = 10, but lid motion is absent at Ri = 1. For a given Ri and Pr, the cavity without a block performed better than the cavity with a square or circular block. The heat transfer coefficient at the heating sources has been established as a performance indicator. Hot source fins improve heat transmission and reduce gallium melting time.

## 1. Introduction

Due to its broad variety of applications, the subject of free convection heat transmission in cavities has been widely researched both computationally and experimentally in recent years. Thermal storage systems, solar power collectors, thermal insulation, heat exchangers, and electronic equipment cooling are well-known examples [1,2,3].

Two proven methods include enhancing free convection heat transmission inside the cavity by adding nanoparticles to the fluid and producing turbulence flow. Because of the fluid’s altered viscosity and thermal conductivity, the presence of nanoparticles in the fluid causes an abnormal treatment of nanofluids [4,5,6,7].

In recent decades, a variety of geometric forms of the cavity have been investigated using different combinations of enforced temperature gradients and cavity topologies. Circles, squares, rectangles, and triangles are the most frequent geometric forms [8,9]. Many scholars have looked at the geometry of triangular, C-shape, concentric annulus, hemispherical, and parallelogrammic shapes for convective heat transport in simple enclosures [10]. Various research on heat transfer analysis in various cavity geometries has been published over the previous decade, culminating in a review paper on the topic.

Previous research using parabolic dish collectors and other cavity receiver geometries and optimization approaches were presented and addressed by Kasaeian et al. (2021) [11]. The work investigates cavity receivers with cylindrical, hemispherical, conical, and flat sides. The conical cavity achieved a thermal efficiency of roughly 70%, energy efficiency of 30%, and optical efficiency of 87% for an inlet temperature of 200 degrees Celsius. Harmand et al. (2013) [12] published a convective heat transfer review in primarily outward airflows in different geometries of a stator–rotor without and with jet impingements, which is divided into two sections: experimental/theoretical approaches and geometries/results. The Geometries and Results section, which proceeds from basic to complicated, explains instances such as a free-spinning disk, a single disk in crossflow, multiple jets, single jet impingement on revolving and stationary disks, and stator-rotor in the systems with and without single jet impingement. Cai et al. (2019) [13] reported research work on the process of complicated cavity ignitions in supersonic flows in five aspects: analysis of non-reacting flows in terms of ignition; the influence of cavity geometry; the influence of ignition technique; the influence of igniter setups; the effect of cavity fueling.

On the other hand, the effects of the aforementioned ignition-influencing factors on the reacting and non-reacting cavity flow fields are investigated. Additionally, common ignition techniques are described in-depth and contrasted in order to illustrate cavity ignition operability in genuine scramjet applications. The solar concentrator performance with various nanofluids and cavity receivers was examined by Loni et al. (2019) [14]. The initial stage of this project is to conduct a thorough evaluation of the research on solar-dish cavity receivers. The second stage is to test the concentrator of the solar dish with a cubical-container receiver utilizing pure thermal oil and Al_2_O_3_/oil nanofluid in an experimental setting. The last part of this project compares the findings discovered with those published in the literature to obtain a broad picture of cavity receivers. Dhaidan et al. (2022) [15] conducted a review of analytical, experimental, and numerical investigations on the solidification of NePCM in cylindrical, planar, spherical, and annular containers. The authors investigated the impacts of dispersed nanoparticle concentrations as well as geometrical and operational characteristics such as wall waviness and heat-transfer fluid flow rate.

Despite this, the literature lacks a full review of heat transport studies in various cavity configurations. As a result, an attempt to conduct this evaluation has been made. This study discusses many technical, research, and development methods for cavity configuration. A solid understanding of the concepts covered will assist future advancements and provide a viable solution for a variety of applications. This research will help researchers understand the varied developments in heat transfer across various cavity geometries, which are still in need of improvement. The current research findings might serve as a roadmap for future studies.

## 2. Review on Circular Cavities

The lid-driven circular cavity may be considered a standard issue for gaining a basic grasp of the system’s internal flow. It has enormous practical as well as academic significance. The lid-driven circular cavity has a wide range of applications, including cooling electronic devices, insulating materials, oil extraction, crystal development, heat exchanger design, float glass manufacturing, painting, food processing, and so on.

**Figure 1:**
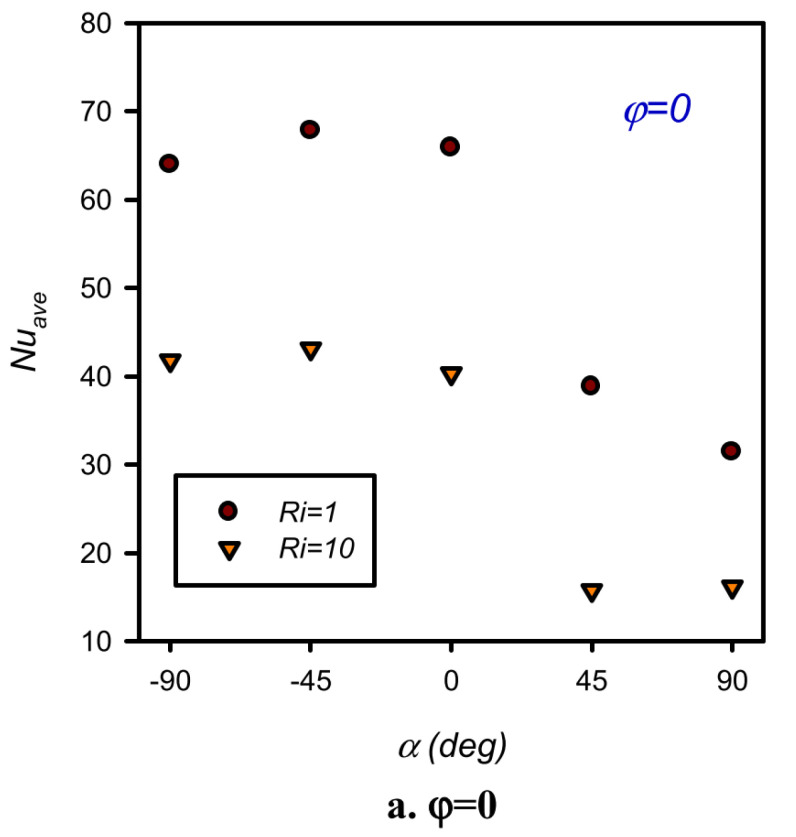
Changes of average Nusselt number with an attack angle of the heated wall for various volume fractions. Reprinted with permission from Ref. [16]. 2019, Elsevier.

Hadavand et al. (2019) [16] studied the impact of the angle of attack, nanoparticle volume percentage, and Richardson number (Ri) on cooling performance and flow phenomena in a cavity of hemi-circular lid-driven. The findings revealed that, by the flow of heating fluids in the passage, heat transmission in the container causes a decline in the temperature of the heated fluid along its path. Moreover, adjusting the attack angle of the cavity causes the heat transfer process to be distinguished, and a varied value of Nusselt number (Nu_ave_) is created for various cases, as illustrated in Figure 1.

**Figure 2:**
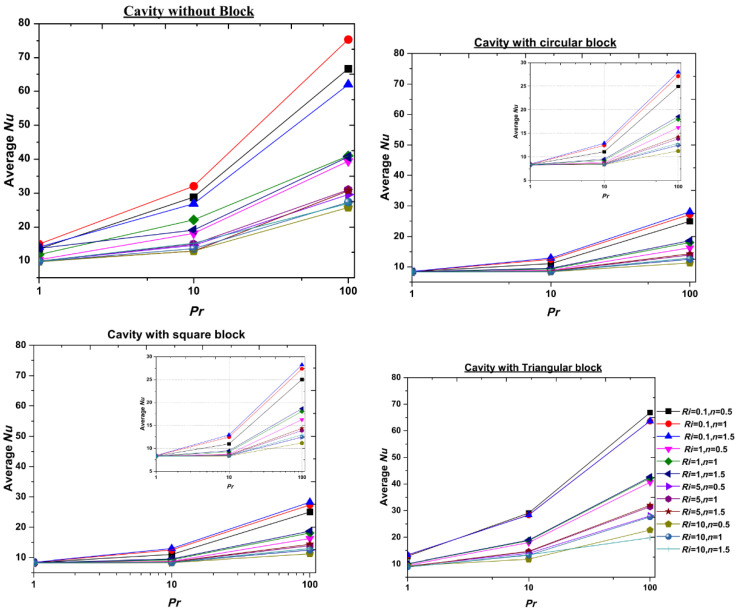
Variation of average Nusselt number with the Prandtl number in a semi-circular cavity with no block. Reprinted with permission from Ref. [17]. 2020, Elsevier.

Gangawane and Oztop (2020) [17] described the thermal and hydrodynamic properties of non-Newtonian power-law fluids in a two-dimensional laminar steady-state. It has been discovered that shear-thickening fluids have greater cooling properties. The horizontal wall/lid is subjected to the ambient thermal condition and slides with a uniform horizontal velocity, whilst the curved surface is subjected to a greater isothermal temperature. Furthermore, as demonstrated in Figure 2, the cavity with an immersed triangle block has superior heat-transmission outcomes than compared with the circular and square blocks.

**Figure 3:**
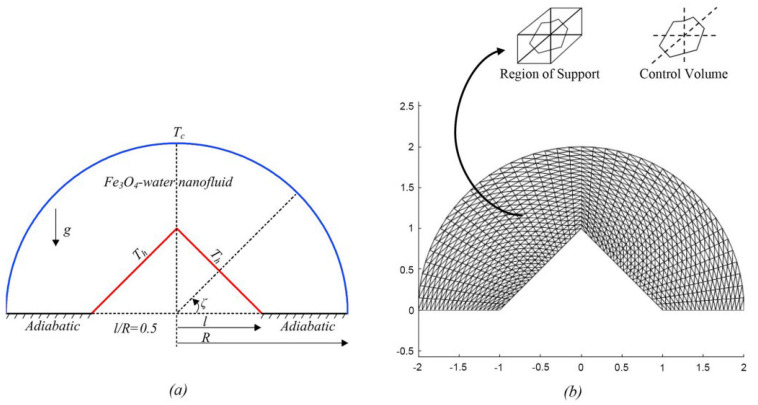
(**a**) Physical model and coordinate system and (**b**) grid distribution. Reprinted with permission from Ref. [18]. 2020, Elsevier.

**Figure 4:**
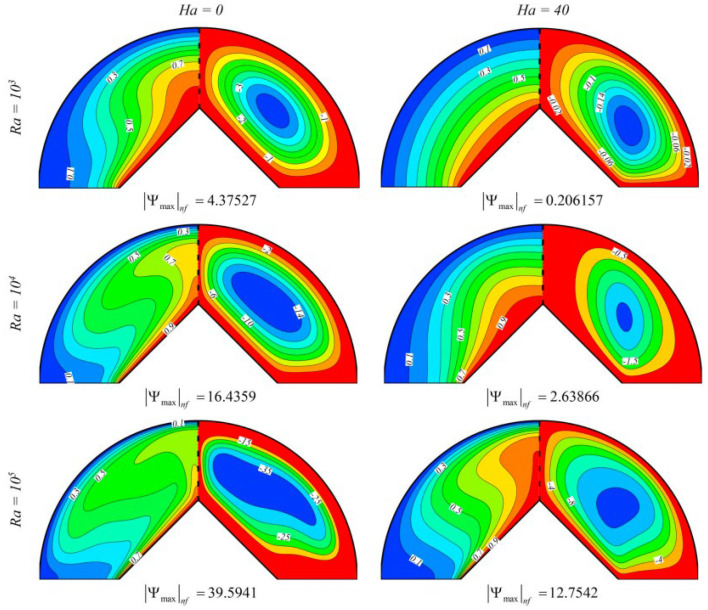
Effect of Ra and Ha on contours of streamlines (**right**) and isotherms (**left**) when ∅ = 2%, Hs = 2. Reprinted with permission from Ref. [18]. 2020, Elsevier.

Using the modified Fourier formula, Dogonchi et al. (2020) [18] investigated the behavior of heat transmission inside a hemi-circular hollow loaded with ferronanofluid (Fe_3_O_4_-H_2_O). Ferrofluid viscosity is calculated using magnetic field-dependent (MFD) viscosity. The nonlinear system is numerically computed by employing the control volume finite element method (CVFEM). According to the findings, the volume percentage of nanoparticles, Rayleigh number, and thermal relaxation factor are related to the average Nusselt number. As observed in Figure 3, spherical nanoparticles have a lower heat exchange rate, while platelet nanoparticles have a higher heat exchange rate. Figure 4 shows the effect of Ra and Ha on contours of streamlines and isotherms when ∅ = 2%, Hs = 2.

**Figure 5:**
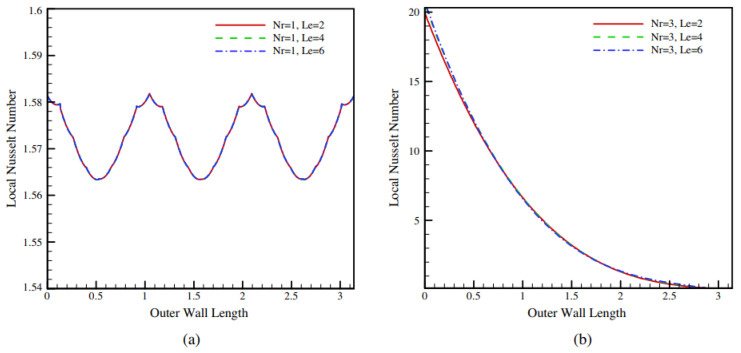
Local Nusselt numbers vs. Le numbers when (**a**) Nr = 1 and (**b**) Nr = 3. Reprinted with permission from Ref. [19]. 2016, Elsevier.

Hatami et al. (2016) [19] examined nanofluid heat transmission in a circular-wavy cavity using natural convection. Based on earlier research, the governing equations for the investigated issue are provided and addressed by employing the finite element technique (FEM). This implies that the amplitude has a greater impact on the heat transmission coefficient than the number of undulations for a wavy wall. The influence of the Le number on the Nusselt number is only noticeable at higher Nr values, and it is almost nonexistent at lower Nr numbers, as observed in Figure 5.

**Figure 6:**
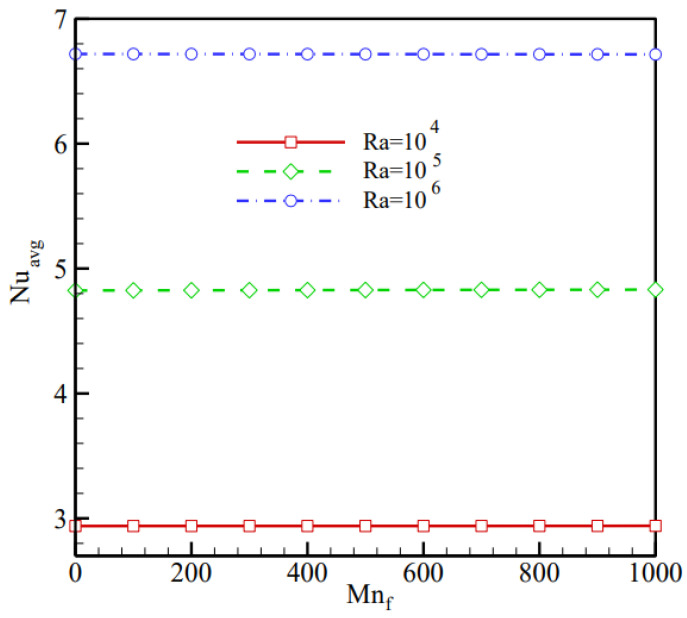
Variation of Nusselt number with the magnetic number for various values of Rayleigh number. Reprinted with permission from Ref. [20]. 2019, Elsevier.

Sheikholeslami et al. (2018) [20] investigated a magnetizable MWCNT-Fe_3_O_4_/H_2_O hybrid nanofluid filling a circular cavity with two circular heaters. Each heater has a wire running through it that transports electrical current. The magnetic fields formed by cables carrying electrical current have different strengths. Hartmann number (Ha), magnetic number (Mn_f_), nanocomposite particle concentration (φ), Rayleigh number (Ra), heater location angle (α), and magnetic strength ratio parameter (γr) are some of the investigated controlling factors. Figure 6 shows how distributing MWCNT-Fe_3_O_4_ hybrid nanoparticles in the host fluid improves convective heat transmission.

**Figure 7:**
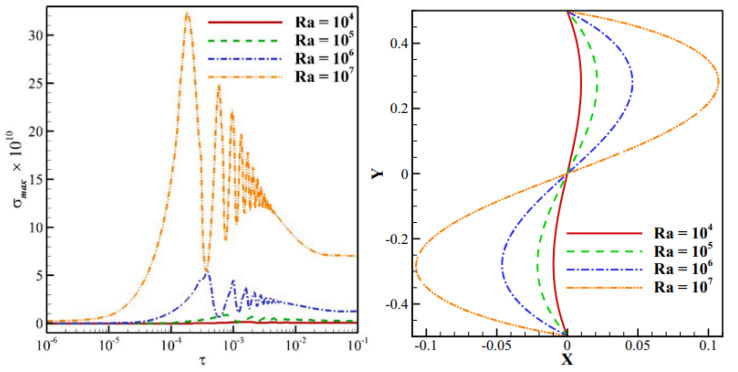
Variation of Rayleigh number with the total stress for the distortion of the movable plate (**left**) and movable plate (**right**) under steady-state conditions. Reprinted with permission from Ref. [21]. 2021, Elsevier.

Ahmadreza et al. (2021) [21] examined the free convection of a Newtonian fluid within a partitioned circular cage with a moveable wall. The Rayleigh number (10^4^ to 10^7^) and the Prandtl number (0.71 to 200) are dimensional metrics that indicate the issue. The findings show that the plate’s deformation degree is proportional to the forces exerted by the fluid. For plate distortion, low Rayleigh numbers and vortex power produced in both cavity parts are nonexistent, and conduction dominates heat transmission. As demonstrated in Figure 7, by increasing the Rayleigh number from 10^4^ to 10^7^, the Nusselt number average will increase by more than five times.

Table 1 presents a summary of review on circular cavities.

According to the review that was detailed earlier related to the circular cavity, it is concluded that the major factor of density difference at Ri = 10 is the cold surface contact with the fluid. Moreover, the host fluid’s Nusselt number increases with MWCNT-Fe_3_O_4_ hybrid nanoparticles. In addition, the block’s presence reduced the cavity’s convection heat transmission.

## 3. Review on Cylindrical Cavities

Cavity structures may be found in a broad range of applications, including electrical equipment and receivers such as a solar cavity. Inhibiting or enhancing heat transmission in cylindrical geometries is required for the improved augmentation of solar cavity receivers or better electronic equipment cooling, prompting engineers to learn more about heat transfer processes in these cavity types.

**Figure 8:**
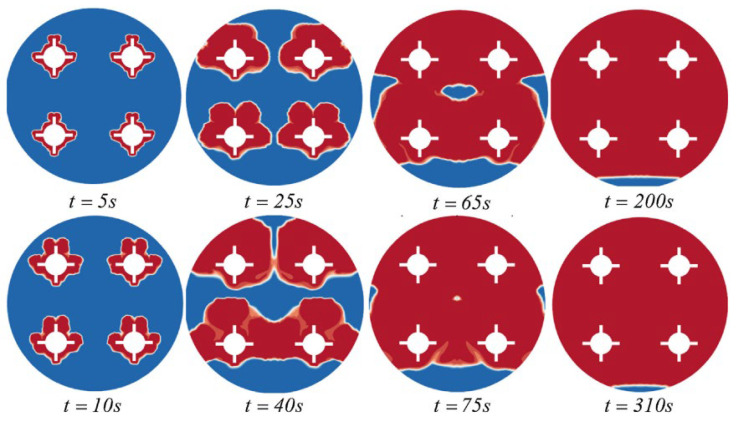
Development of the liquid fraction of heating sources with fins (Th = 40 °C). Reprinted with permission from Ref. [22]. 2018, Elsevier.

**Figure 9:**
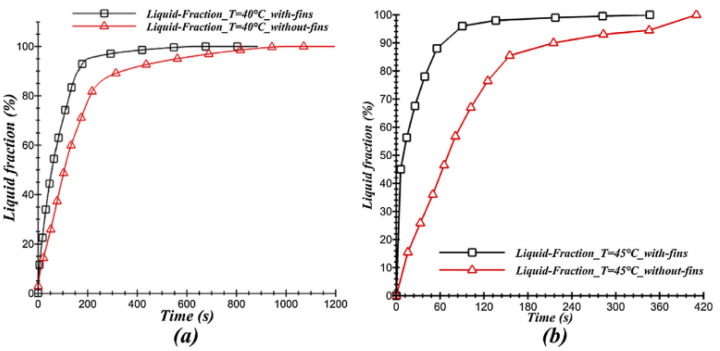
Development of liquid fraction of the studied configurations for (**a**) T_h_ = 40 °C and (**b**) T_h_ = 45 °C. Reprinted with permission from Ref. [22]. 2018, Elsevier.

Bouhal et al. (2018) [22] used computational fluid dynamics simulation to study the melting of PCM within a cylindrical-shaped enclosure with heating sources. The CFD model with the formulation of physical enthalpy-porosity is applied to simulate solid Gallium phase transition and to optimize the heating source according to operating conditions and applied temperatures. Fin and cylindrical heating sources were investigated at two distinct temperatures (Th = 40 and 45 °C). Figure 8 and Figure 9 show that the cylindrical chamber with four fins incorporated at each heating source increased heat transmission and melting time in the PCM.

**Figure 10:**
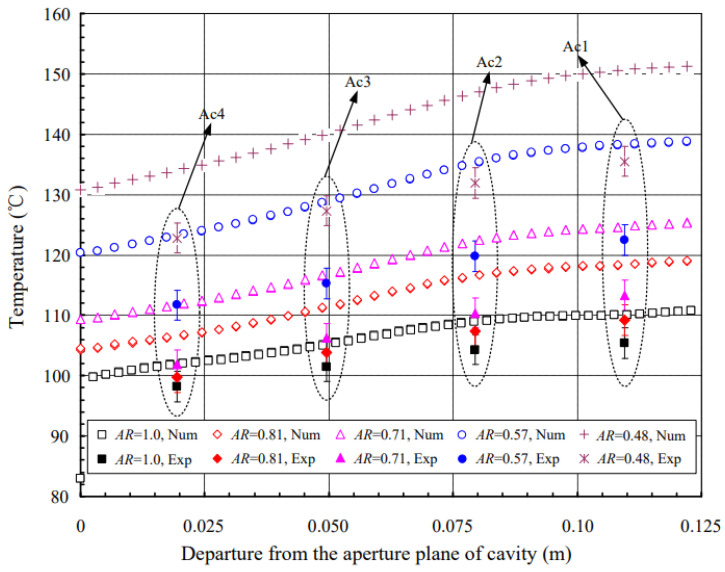
Temperature variation with departure from the aperture plane of the cavity. Reprinted with permission from Ref. [23]. 2017, Elsevier.

Shen et al. (2017) [23] studied the impact of the size of the aperture on radiation heat transmission and free convection in the upward-facing cylindrical cavity with isoflux. Experiments were also carried out to examine the impacts of heat flux and tilt angle for various aperture diameters. According to the results, the larger the size of the aperture, the cooler the cavity surfaces and the more efficient the heat transfer by radiation and free convection. Figure 10 shows that convection heat transmission is affected by aperture size, whereas in radiation heat transfer, the reverse occurs.

Shen et al. (2015) [24] studied the effects of the cavity’s surface heating condition, heat flow, and tilt angle on combined-radiation heat transmission and free convection in an upward-facing cylindrical enclosure. In light of this intriguing finding, a three-dimensional numerical analysis was carried out, which was supported by relevant experimental data, to study the influence of the tilt angle of the cavity from the standpoint of the physical process. The authors provided an empirical correlations of Nu_c_ and Nu_r_ under three surface heating settings using cavity tilt angles and heat flux as independent factors.

Shen et al. (2016) [25] used a cylindrical chamber with a constant heat flow. Following that, the notion of the tilt angle of the critical cavity was proven, and its value was established using three-dimensional models of unstable and stable simulation. The confirmed steady-state model was used to explore the impacts of heat flux and aperture ratio on the tilt angle of the critical cavity without sacrificing computation cost and accuracy. The significance of choosing proper monitoring parameters was also discussed in depth. The authors concluded that the critical tilt angle of a partly open cavity rises with rising heat input or decreasing aperture ratio.

A non-isothermal fluid in a rotating cavity was explored computationally and experimentally by Vjatkin et al. (2019) [26]. A revolving horizontally mounted cylinder with isothermal boundaries exposed to translational vibrations perpendicular to the rotation axis was used to study heat-generating fluid convection. The average heat transfer and convection increase when the vibration frequency approaches the rotation frequency. It was also noticed that the vibrations disrupt the centrifugal force field’s axial symmetry when the frequencies coincide. As a consequence, the temperature at the cavity axis is significantly reduced.

Xiao et al. (2020) [27] experimentally examined and analyzed the characteristics of natural convective heat loss for a cylindrical container without and with a quartz window. By varying the heated states and tilt angles of the walls, the thermal performance of two kinds of cavity receivers is investigated, and the influence of the quartz window on the cavity’s heat loss is emphasized. The finding is that the quartz window with an average cavity temperature is 68.61 °C, 79.36 °C, and 48.22 °C higher than the identical instances without a quartz window (corresponding tilt angle is −90°, −30°, and 45°). The experimental findings demonstrate that the quartz window may greatly raise the cavity’s operating temperature and reduce the tilt angle’s impact.

**Figure 11:**
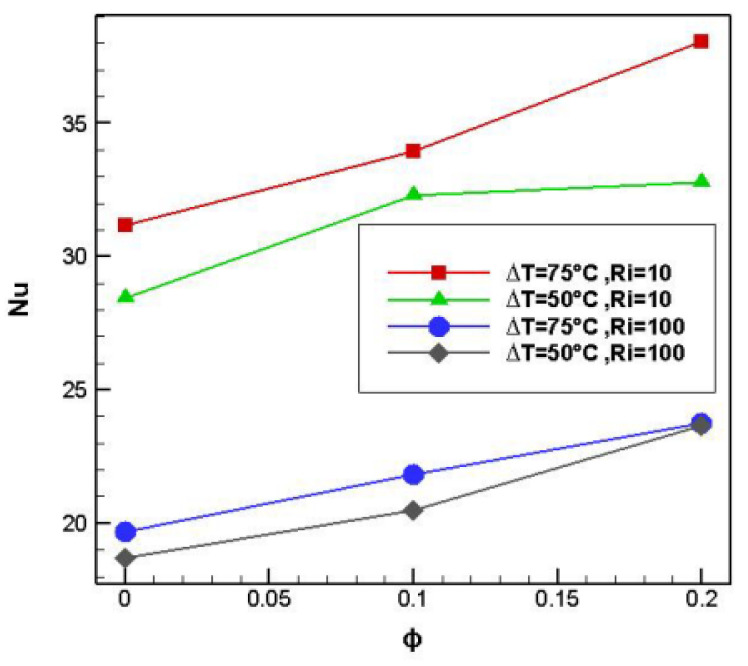
Nu versus ϕ in different ΔT and Ri. Reprinted with permission from Ref. [28]. 2019, Elsevier.

Al-Rashed et al. (2019) [28] investigated mixed nanofluid convection using aluminum oxide and water nanoparticles in a lid-driven cavity with a heated elliptical central cylinder. As illustrated in Figure 11, Nusselt number and heat transfer rose as the temperature differential between the lower temperature surfaces and the hot cylinder increased, as did Richardson number and solid volume percentage. The gained findings reveal that when the solid volume percentage increases, more nanofluid condensation occurs, and more Brownian motion occurs.

**Figure 12:**
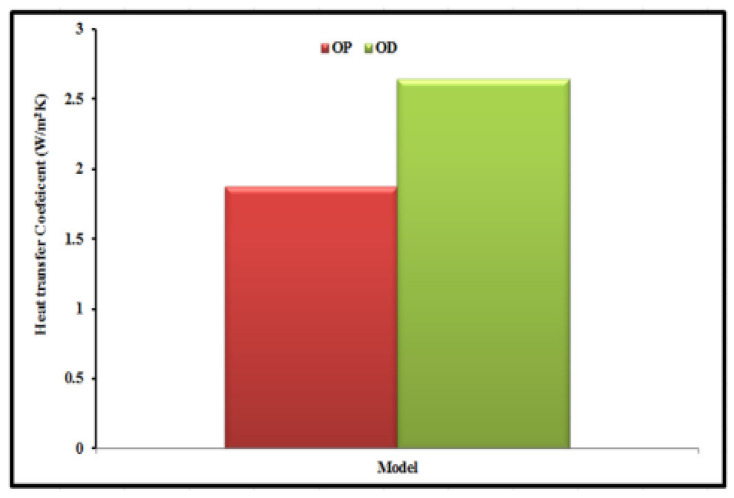
The transfer coefficient for the two configurations of the cylindrical receiver. Reprinted with permission from Ref. [29]. 2019, Elsevier.

The influence of the cylindrical cavity’s thermal-receiver shape on the performance of a small-scale solar Brayton cycle application was investigated by Daabo et al. (2019) [29]. It also calculates the best form for the receiver setup under investigation. They also work on optimizing a small-scale cylindrical form. The receiver form was produced using surface optimization and CFD analysis methods, using the ANSYS Workbench software program, with the aim of reducing the heat-loss convective mode. The internal height of the receiver and the two borders on the bottom receiver’s width, as shown in Figure 12, were the most influential parameters on the value of the heat transfer coefficient.

**Figure 13:**
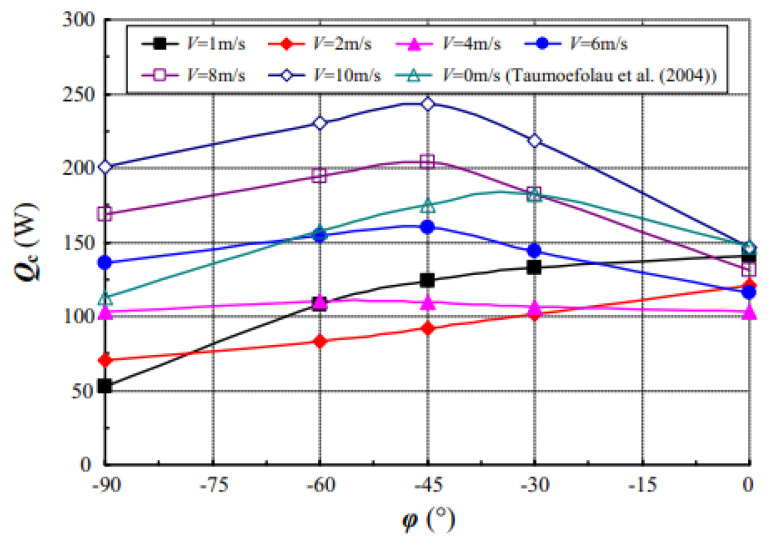
Dependence of Q_c_ on φ for various V at α = 90° and T_W_ = 923 K. Reprinted with permission from Ref. [30]. 2016, Elsevier.

By using a three-dimensional numerical model, Shen et al. (2016) [30] investigated an isothermal upward-facing hollow cylinder as the target under windy conditions and coupled convective heat loss. Wind speed, surface temperature, the tilt angle of the cavity, and wind incidence angle all had an impact. According to the findings, increasing the surface temperature enhances convective heat loss. Almost every time the speed of wind rises from zero, the total convective heat loss decreases at first and then increases. Moreover, in no-wind conditions, the minimum is significantly lower, as observed in Figure 13.

**Figure 14:**
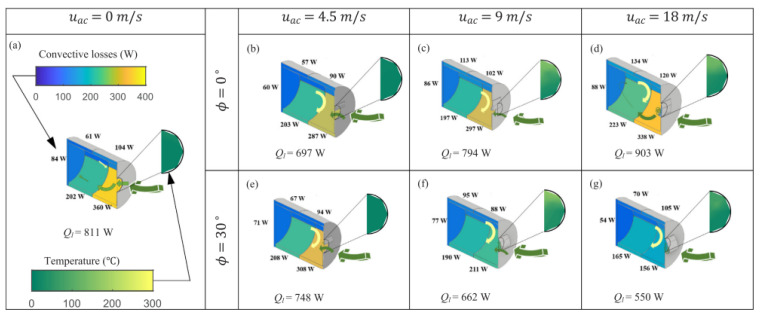
Color maps of the surface distribution over the temperature of the air and measured convective heat losses in the aperture plane for a series of conditions. (**a**) uac = 0 m/s; (**b**) uac = 4.5 m/s, ϕ = 0°; (**c**) uac = 9 m/s, ϕ = 0°; (**d**) uac = 18 m/s, ϕ = 0°; (**e**) uac = 4.5 m/s, ϕ = 30°; (**f**) uac = 9 m/s, ϕ = 30°; (**g**) uac = 18 m/s, ϕ = 30°. Reprinted with permission from Ref. [31]. 2020, Elsevier.

Alipourtarzanagh et al. (2020) [31] evaluated the effectiveness of the air curtain in reducing losses of convective heat from a heated receiver of a cylindrical cavity operating at yaw angles of (0°) and a specified tilt angle (15°). The chamber was electrically heated to a constant temperature of 300 °C. The impacts of discharge angle, variable wind speed, and air curtain velocity were investigated. It was discovered that raising the velocity of the air curtain with a discharge angle of 0° results in increased convective heat losses. Increasing the air curtain velocity by 60% may minimize heat losses by up to 60% for a 30°as discharge angle of the curtain, as shown in Figure 14.

Zhang et al. (2022) [32] suggested integrating a new fin structure with Al_2_O_3_ nanoparticles to increase the melting performance of PCMs for thermal energy storage devices. A mathematical model of PCM melting with nanoparticles in a triple-tube heat exchanger was developed and verified. The findings show that new fins and nanoparticles improve melting. The melting time of four new fins is lowered by 80.35 percent, 77.62 percent, 77.33 percent, and 80.65 percent, respectively, compared to the original structure. Al_2_O_3_ nanoparticles (3, 6, and 9 percent) incorporated into PCMs’ lower melting time by 13.1%, 15.6%, and 18.8%, respectively.

Bouzennada et al. [33] provided a 2D-symmetric numerical analysis of a novel NEPCM-filled enclosure design. The enclosure features a tube for circulating heat transfer fluid (HTF); n-Octadecane is used as phase change material (PCM). Comsol-Multiphysics solves the governing equations. The inner tube had three vertical orientations, and the nanoparticle concentration was 0 to 0.06. Both heat transfer/melting speeds are enhanced when the inner tube is near the bottom of the enclosure and the nanoparticle concentration increased.

Phase change material (PCM) was suggested by Han et al. (2022) [34] as a thermal energy storage unit to assure the stability and adaptability of solar-powered heating and cooling systems. The PCM melting process is evaluated using a mathematical model that incorporates the influence of nanoparticles on heat transport. Even at the earliest stage of PCM melting, spontaneous convection owing to the buoyancy effect dominates the flow’s behavior, as shown by the data. High natural convection at the base of the annular tube pulls the liquid–solid boundary lower by moving the molten PCM upwards from the side. The inclusion of Al_2_O_3_ nanoparticles at a concentration of 3 percent by volume improves PCM melting performance by shortening PCM melting time by roughly 15 percent. Comparing Al_2_O_3_, copper, and graphene nanoparticles indicates that an increase in thermal conductivity does not significantly enhance the melting ability of PCMs.

Table 2 provides a summary of review on cylindrical cavities.

According to the outcomes of the above works related to the cylindrical cavity, it is concluded that the fin design increased heat transmission and sped up the melting time of the PCM. Moreover, the changing patterns of radiation heat transfer Nusselt numbers and free convection with heat flux and tilt angle are unaffected by aperture size. In addition, transverse vibrations are effective tools for controlling heat convection in spinning systems.

## 4. Review on Hexagonal Cavities

In CFD, convection heat transport within cavities of various geometries is focus of research. The food processing industry, industrial processes, mining operations, solar collectors, the electronics industry, phase transition materials, nuclear plants, and other engineering and scientific applications are only a few examples. Due to their vast uses, flows within hexagonal cavities have received the greatest attention as critical infrastructure for coastal and offshore engineering.

Ahmed and Raizah (2021) [35] investigated the law of non-Newtonian power nanofluid flow between inclined polygonal–cylinder polygonal–polygonal forms using the second law of thermodynamics. Electromagnetic forces, as well as thermal radiation, are considered. Ethylene glycol (as a base fluid) was mixed with multi-wall carbon nanotubes to construct the working suspension. The major findings indicated that the convective and heat transfer processes governed the flow area and power-law index. Moreover, the fluid friction’s irreversibility peaks at large power-law index values.

**Figure 15:**
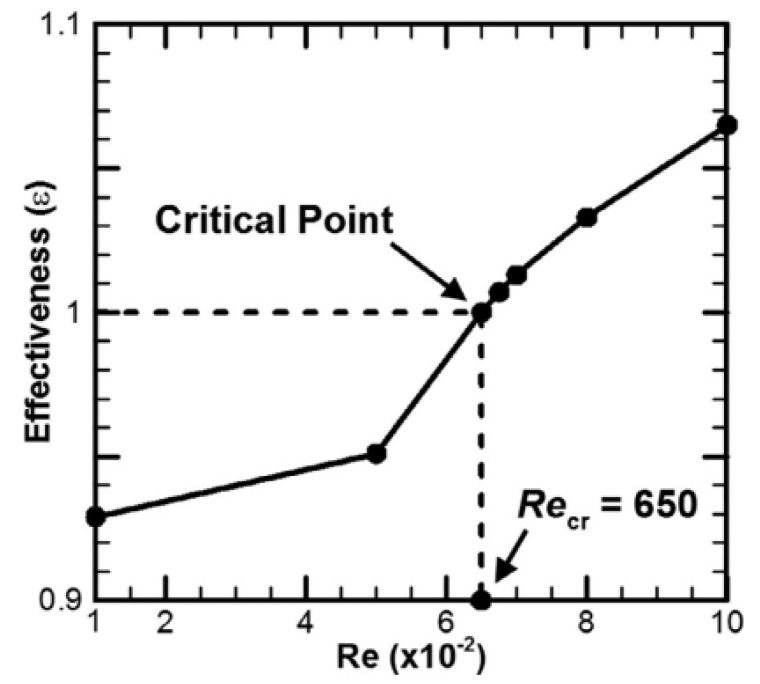
Dependence of heat transfer effectiveness on Reynolds numbers. Reprinted with permission from Ref. [36]. 2021, Elsevier.

Ikram et al. (2021) [36] presented the unsteady analysis of heat transfer conjugate forced convection and flow characteristics in the air-filled hexagonal cavity. Under the rotating effect of an adiabatic flow modulator, this container was supplied with a continuous heat flux heater. Furthermore, the blade is positioned in the cavity’s middle point, which rotates clockwise. As observed in Figure 15, the heat transfer efficiency is inversely proportional to the Biot and Rayleigh numbers but increases dramatically as the Reynolds number rises.

**Figure 16:**
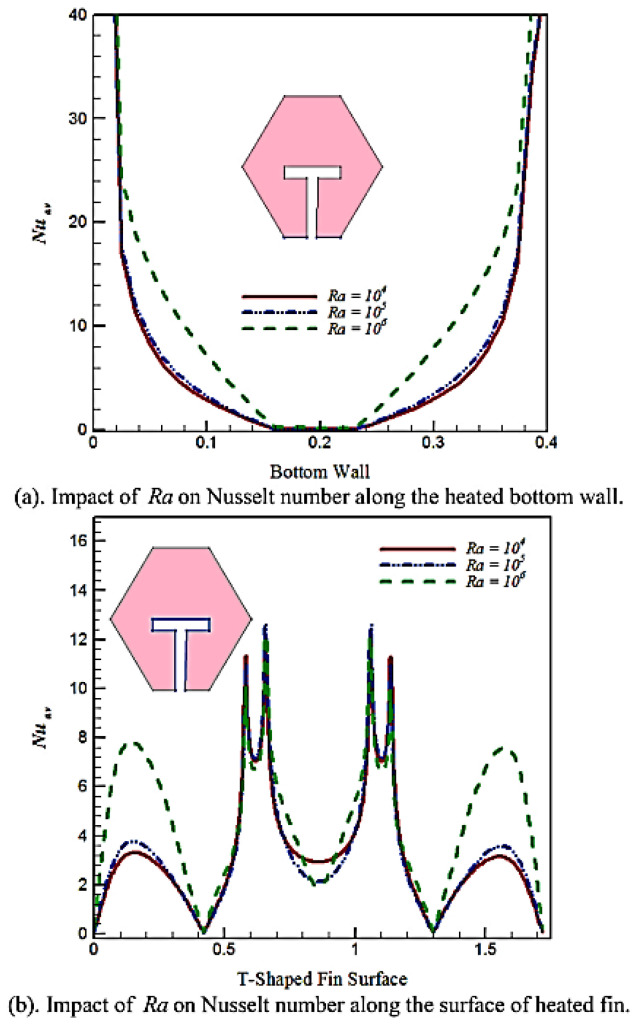
Impact of Ra on Nusselt number. Reprinted with permission from Ref. [37]. 2020, Elsevier.

Rehman et al. (2020) [37] reported a numerical analysis of a buoyantly convective non-Newtonian fluid flow in a hexagonal-shaped cavity. The consistently heated T-shaped fin is inserted in the hexagonal cavity’s bottom wall. Both triangular and rectangular components are used to discretize the hexagonal cavities as a computing domain. The distribution of temperature and velocity around an evenly heated T-shaped is investigated in terms of the Rayleigh number. When the Rayleigh number increased, the heat transfer rate over the surface of the T-shaped fin increases, as observed in Figure 16.

**Figure 17:**
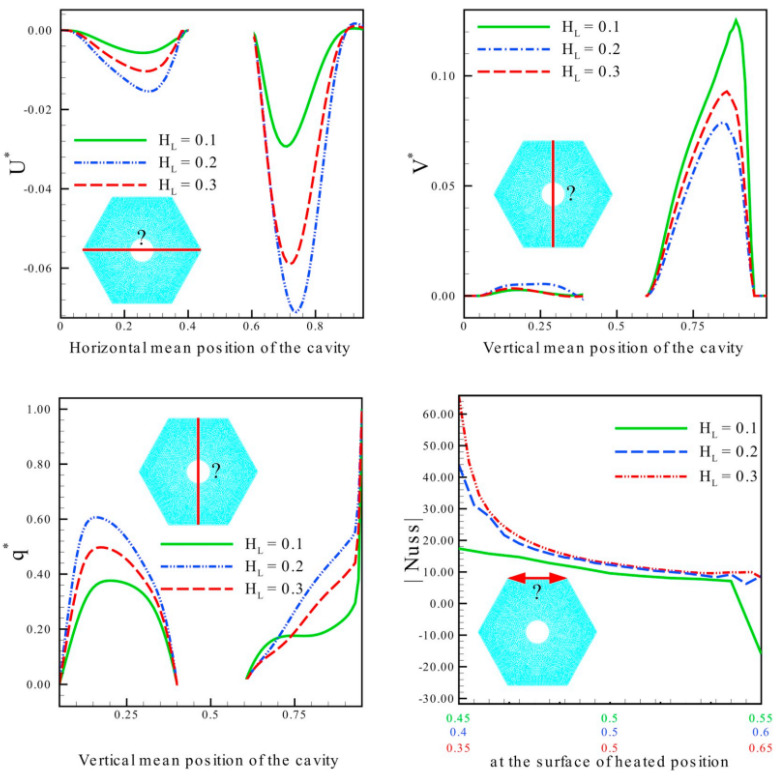
Velocities and temperature variation at mean Nusselt number and position. Reprinted with permission from Ref. [38]. 2020, Elsevier.

Haq et al. (2020) [38] provided a numerical investigation of the lid-driven hexagon hollow filled with water. The top wall of the hollow is partly heated and flowing at a uniform velocity. Furthermore, a cylindrical barrier is placed within a hexagonal hollow, imposing various constraints on the surface. The simulation is run to estimate the heat transfer rate, steam lines, and isotherm behavior as a function of the physical and geometrical factors included in the model, including Reynolds number, Richards number, Hartman number, and heated length. As shown in Figure 17, the circular obstacle played an important part in the development of isotherms by changing the different constraints at the surface.

**Figure 18:**
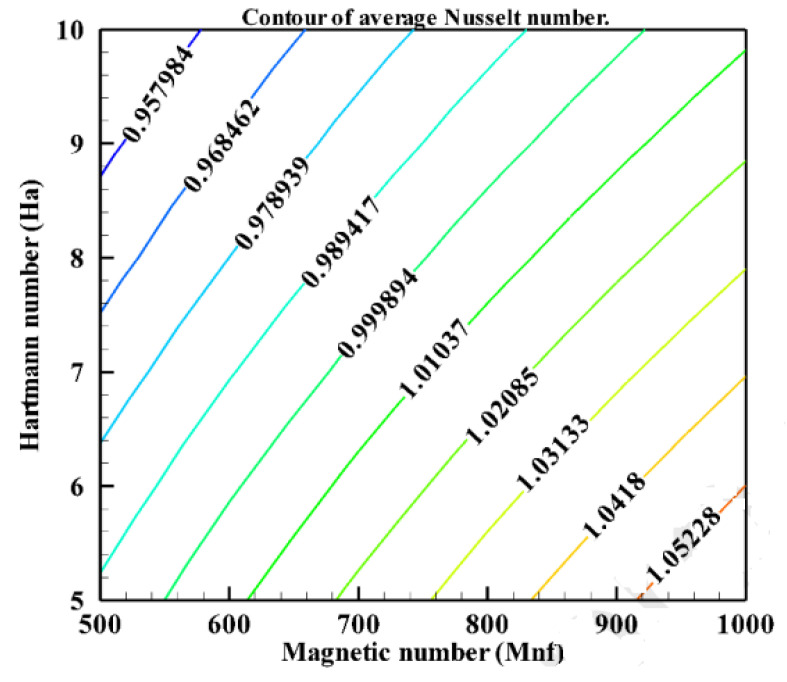
The contours of the average Nusselt number in Ra = 10^3^ for different magnetic and Hartmann Numbers. Reprinted with permission from Ref. [29]. 2020, Elsevier.

Rizwan et al. (2020) [39] performed numerical simulations in a water-filled hexagon hollow. The top wall of the hollow is uniformly heated. A cylindrical obstruction is also placed within a hexagonal hollow, establishing surface constraints. The simulation is run to predict the rate of heat transfer, isotherms behavior, and steam lines as a function of the Reynolds, Richards, Hartman, heated length, and cylindrical. As observed in Figure 18, the circular obstruction helped produce isotherms by altering surface constraints.

**Figure 19:**
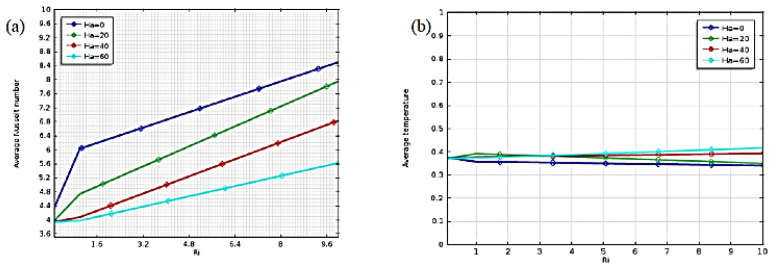
Effect of Hi and on (**a**) mean Nusselt number and; (**b**) mean temperature. Reprinted with permission from Ref. [40]. 2017, Elsevier.

Alia et al. (2017) [40] studied the magnetohydrodynamic behavior of mixed convection heat transmission in a hexagonal hollow. The enclosure’s horizontal walls are kept at a consistent temperature while the inclined walls are heated evenly. A variety of Richardson numbers (0.1 to 10) and Hartmann numbers (0 to 60) are shown visually as streamlines, isotherms, average Nusselt number, and average temperature. As observed in Figure 19, the Hartmann and Richardson numbers significantly impact the temperature field and flow shape.

**Figure 20:**
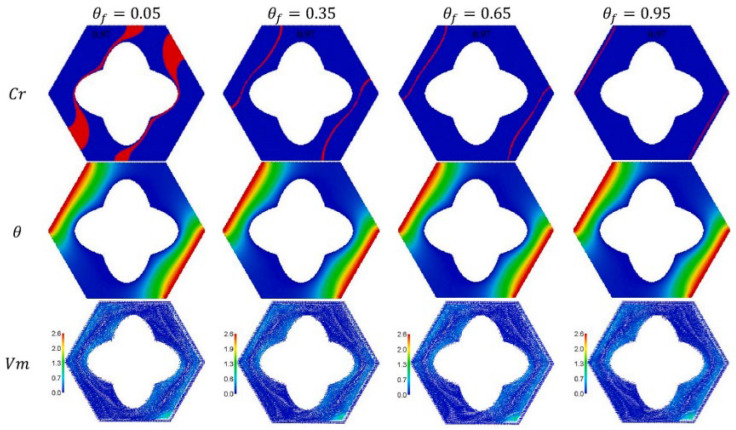
Under changing fusion temperatures θ_f_, velocity field, the heat capacity, and isotherms. Reprinted with permission from Ref. [41]. 2021, Elsevier.

Using the ISPH approach, Aly et al. (2021) [41] explored the effect of magnetic fields on thermosolutal convection of nano-enhanced PCM in a porous annulus. A unique annulus is formed by an outside hexagonal and a dual interior curve of variable lengths. The mass/heat source is on the right bottom and left of a hexagonal cavity, while the rest is adiabatic. As observed in Figure 20, increasing the fusion temperature pushes the zone of phase change away from the dual curves and towards the locations of the heater source.

**Figure 21:**
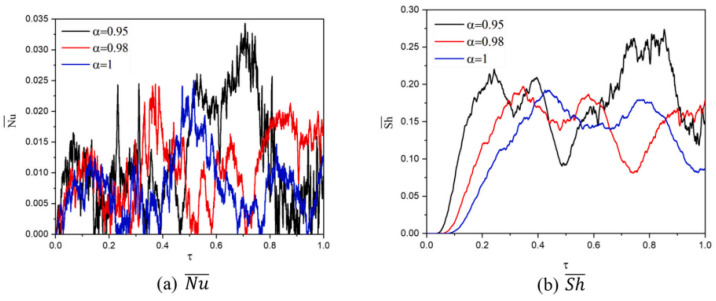
Profiles of $\bar{Nu}$ and $\bar{Sh}$ with fractional derivative parameter (α). Reprinted with permission from Ref. [42]. 2021, Elsevier.

Aly et al. (2021) [42] simulated thermosolutal convection of NEPCM inserted in an annulus between an exterior hexagonal-shaped hollow and an inner wavy form. The mass and heat transmission of NEPCM inside an annulus was altered by two rotations between the inner wavy and outer hexagonal cavities. Reducing fractional derivative parameter α from 1 to 0.95 improves maximum nanofluid velocity by 13.73%, as observed in Figure 21.

Table 3 shows a summary of review on hexagonal cavities.

According to the outcomes of the above works related to the hexagonal cavity, it is concluded that Ra increases heat capacity, temperature, and concentration, and Cr curves increase over an annulus. Moreover, the increased ratios of entropy tool values due to the variations of *γ* take their high values at minimum values of Ha and *γ* = 30. In addition, the double rotations accelerated heat capacity, nanofluid flow, concentration inside an annulus, and altered temperature.

## 5. Review on Rectangular Cavities

In different technical fields, such as PV panels, cooling, electrical components, heat transfer in building envelopes, solar collectors, and natural convection flow occur in rectangular chambers with both ends accessible to the external environment.

**Figure 22:**
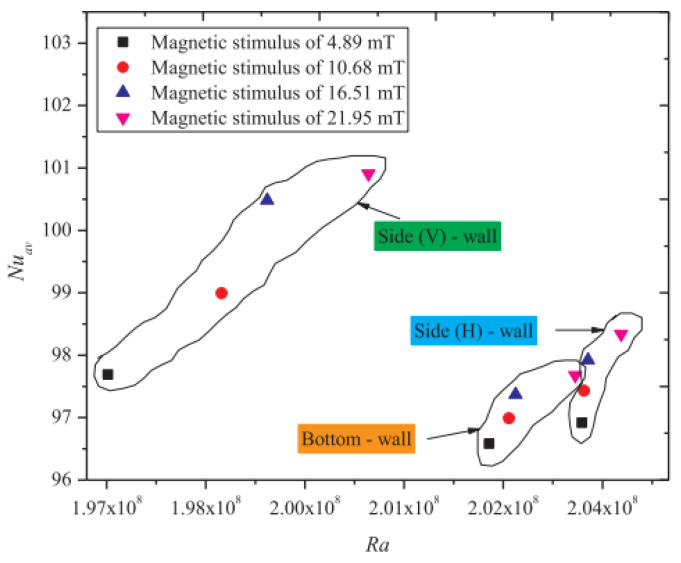
Nu_av_ enhancement with increasing magnetic induction. Reprinted with permission from Ref. [43]. 2020, Elsevier.

Giwa et al. (2020) [43] studied the free convection heat transport of aqueous Fe_2_O_3_-Al_2_O_3_ (75:25) nanofluids in a rectangular cavity. To induce buoyancy, the two opposing vertical walls were exposed to gradients ranging in temperature from 20 to 35 °C. The increase in the magnetic field strength from 48.9 to 219.5 G improved heat transmission by 0.10 vol.% AHF. As observed in Figure 22, hybrid nanofluids outperform nanoparticle nanofluids in heat transmission.

**Figure 23:**
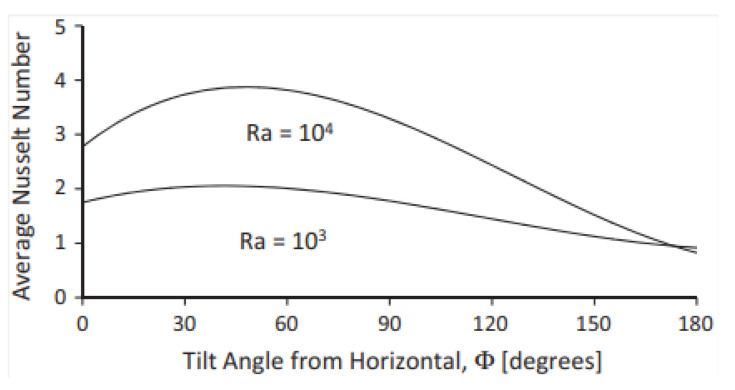
Variation of average Nusselt number with tilt angle, ϕ. Reprinted with permission from Ref. [44]. 2015, Elsevier.

The aspect ratio and tilt angle of inclined rectangular chambers with two localized heat sources were studied numerically by Elsherbiny and Ismail (2015) [44]. The mass, momentum, and thermal energy equations were solved mathematically. The study employed rectangular cavities with covered Rayleigh numbers s/A ranging from 10^3^ to 10^6^, heater position ratios of B1 = 0.25, B2 = 0.75, and size ratio ϵ = 0.25. The findings are graphed as isotherm and streamline contour plots. Figure 23 shows the heat transport parameters and average Nusselt values.

**Figure 24:**
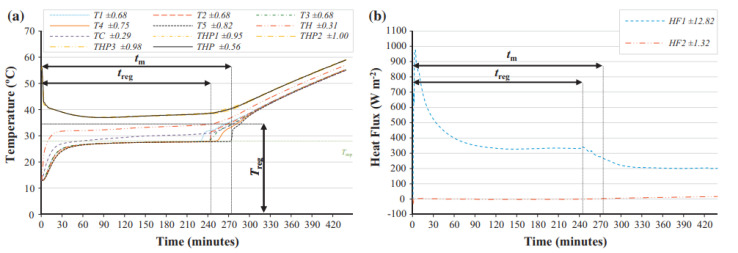
Evolution of the average measured (**a**) temperatures and (**b**) heat fluxes for the 34 W power level charging phase of the 5 cavities test sample filled with the free-form PCM-RT 28 HC. Reprinted with permission from Ref. [45]. 2015, Elsevier.

Soares et al. (2015) [45] investigated heat transport via a vertical stack of rectangular cavities filled with PCMs during melting and solidification. The free-form PCM-Rubitherm RT 28 HC and the microencapsulated PCM-MicronalDS 5001 X are studied. The primary purpose is to debate whether the PCM type is preferable for constructing applications. It examines the temperature used in controlling the heated surface and the time taken for regulation. As illustrated in Figure 24, natural convection improves both characteristics of the PCM for the thermal management of vertical layouts.

**Figure 25:**
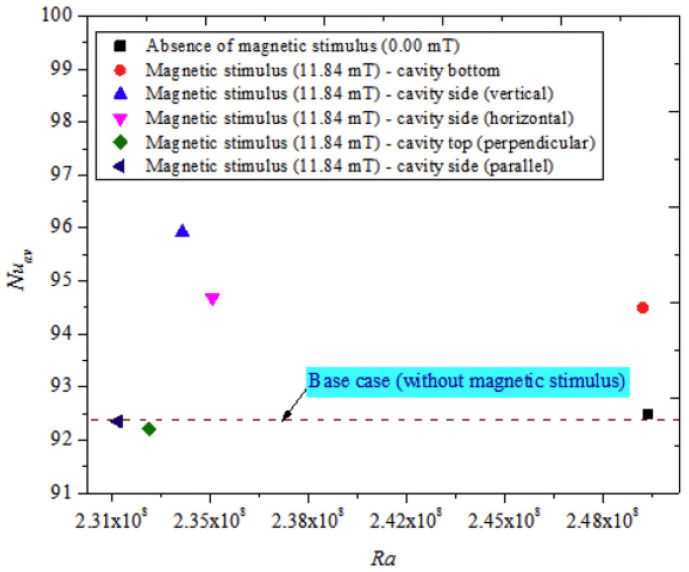
Variation of magnetic stimulus with Nu_av_ for different cavity walls. Reprinted with permission from Ref. [46]. 2020, Elsevier.

An experimental investigation on the dual impact of hybrid nanofluid and magnetic excitation on thermo-convection characteristics in enclosures was addressed by Giwa et al. (2020) [46]. Thermo-convection heat transfer behavior was studied by utilizing unique Fe_2_O_3_-MWCNT (80:20)/deionized water nanofluids (φ = 0.05 to φ = 0.4 vol.%). The HMNFs’ stability and morphology were investigated. The cavity was charged with DIW and stable HMNFs and heated to 20–35 °C. (Ra), and ΔT increased without magnetic stimulation, as shown in Figure 25.

**Figure 26:**
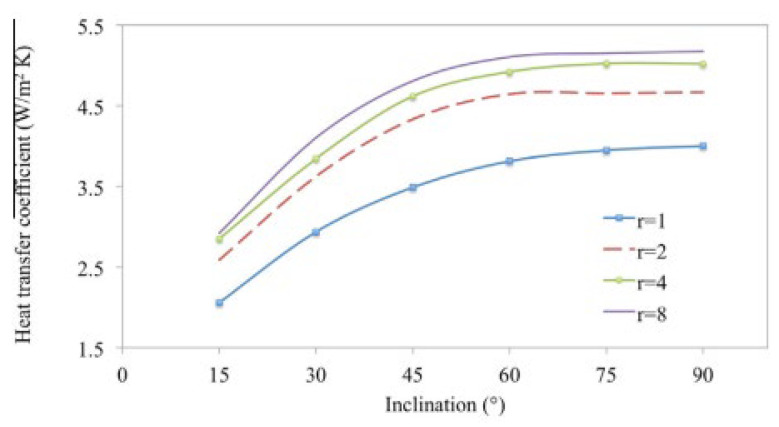
Heat transfer coefficient variation in inclined rectangular cavities. Reprinted with permission from Ref. [47]. 2016, Elsevier.

Li and Tong (2016) [47] investigated natural convection heat transmission in low-width-to-height inclined rectangular chambers. To simulate convective heat transfer and airflow movement in the inclined rectangular chamber, a three-dimensional (3-D) model was first constructed using the CFD ANSYS-Fluent. Improved convective heat transmission and the encouragement of free convection flow in the inclined rectangular cavity are depicted in Figure 26.

**Figure 27:**
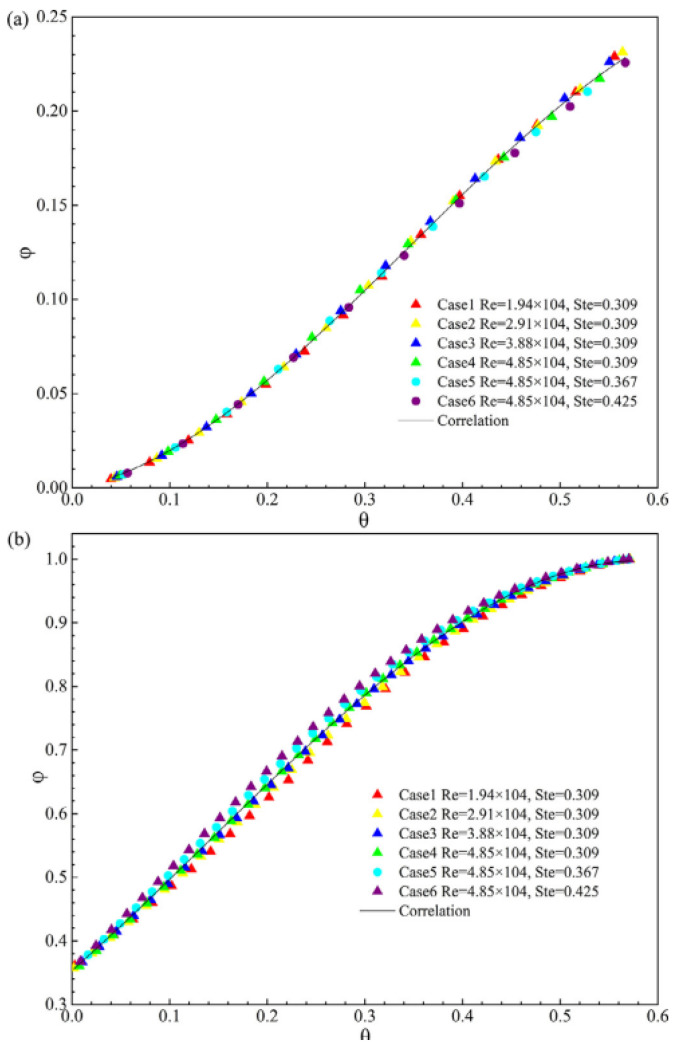
Overall results for melting fraction against θ with different Stefan numbers and Reynolds numbers in the first stage (**a**) and second stage (**b**). Reprinted with permission from Ref. [48]. 2020, Elsevier.

Qi et al. (2020) [48] numerically investigated PCM’s melting process and the thermal behavior in a rectangular container with one side that was thermally insulated and HTF flowing over the other. PCM and HTF involve lauric acid and hot air in this study. The findings reveal that despite the decreasing heat transmission rate through the cavity wall, the melting process is comparatively high in the cavity’s top half due to natural convection. Unlike in tube flows, increasing the Reynolds or Stefan numbers considerably benefits the melting process, as observed in Figure 27.

**Figure 28:**
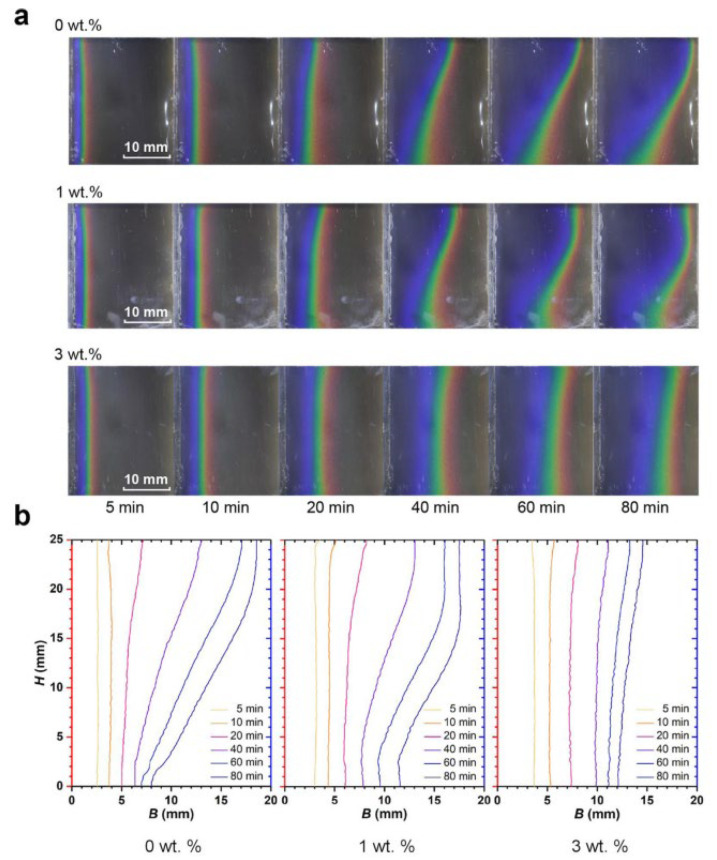
Phenomenological changes in the H/B = 1.25 rectangular cavity during melting of several NePCM samples: (**a**) TLC thermography snapshots collected at representative times and (**b**) comparison of the thermal images’ melting front. Reprinted with permission from Ref. [49]. 2020, Elsevier.

For the first time, Li et al. (2020) [49] employed thermochromic liquid crystal (TLC) to image the unseen phase boundaries of NePCM. The cavity height-to-width aspect ratios were 0.8 and 1.25. In the upper and lower sections of the hollow, free convection from the top corner generated a variance in melting patterns. The lack of natural convection causes heat conduction to dominate during melting, as observed in Figure 28.

Thiers et al. (2020) [50] identified the best thermal disturbance parameters to improve heat transmission in a four-aspect-ratio rectangular differentially heated cavity. Therefore, the influence was computed from 1 to 2 tiny thermal actuators on heat transmission in this kind of cavity. Flows are estimated by programming the 2D equations of unsteady Boussinesq–Navier–Stokes. This enhancement reaches 5.5% by disrupting both local synchronized square waves with active walls of frequency f = 0.403 and amplitude *ε* = 1.

**Figure 29:**
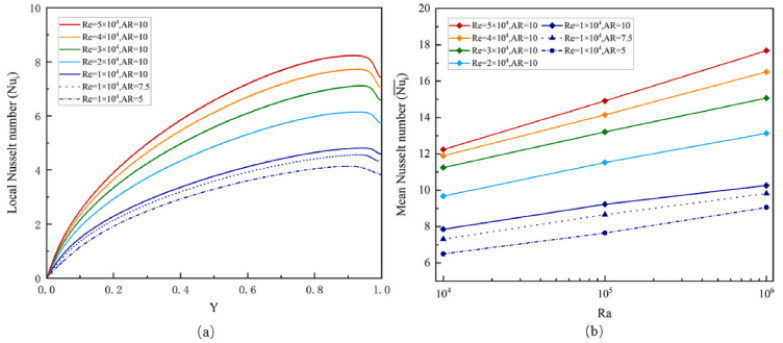
Variation of (**a**) local Nusselt number and (**b**) mean Nusselt number of the mPCM. Reprinted with permission from Ref. [51]. 2021, Elsevier.

Li et al. (2021) [51] examined the pattern of convective heat transfer of mPCM in a narrow rectangular container with continuous heat flux and flow boundary conditions. The impacts of Rayleigh, Reynolds, and aspect ratio on mPCM’s convective heat transport processes are studied. As observed in Figure 29, raising Ra or Re improves convective heat transfer efficiency.

**Figure 30:**
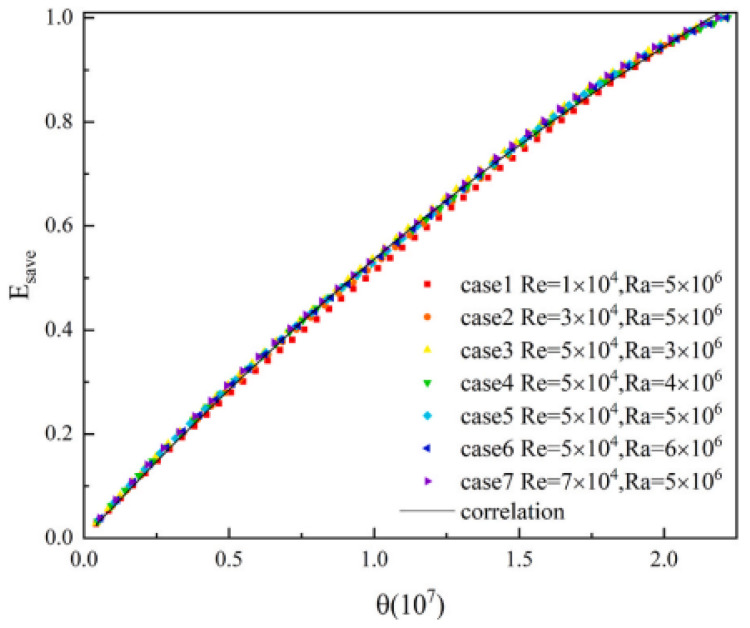
Fitting curves to thermal energy storage fractions in all situations. Reprinted with permission from Ref. [52]. 2021, Elsevier.

Qin et al. (2021) [52] explored the numerical melting of PCM in a container of rectangular geometry with HTFs flowing from each side. The findings reveal that the cooling between liquid PCM and solid–liquid interface and the non-uniform heat transmission of HTF caused natural convection. The boundaries of asymmetric flow affected the PCM melting process, heat transport, and thermal energy storage. As observed in Figure 30, the melting process is separated into three phases.

Table 4 shows a summary of review on rectangular cavities.

According to the outcomes of the above works related to the rectangular cavity, it is concluded that the Reynolds and Rayleigh numbers play the main roles in melting PCM. Moreover, the amount of cold and hot fluids combined in the cavity influence local heat transfer at the wall. In addition, the optimal area for improving heat transmission is 70% of the hot plate height or 30% of the cold plate height.

## 6. Conclusions

This paper examines the latest developments in heat transfer analyses in various cavity shapes. The impact of operating factors and the creation of different cavity designs are examined. The key results are as follows:

1. The Nusselt number graphs behave differently when decreasing the Richardson number.
2. Comparatively, the natural heat transfer process dominates at Ri = 10, but lid motion is absent at Ri = 1.
3. For a given Ri and Pr, the cavity without a block performed better than the cavity with a square or circular block.
4. As a performance indicator, the heat transfer coefficient at the heating sources has been established. Hot source fins improve heat transmission and reduce gallium melting time.
5. Increasing the aperture size boosts both radiation and free convection heat transfer.
6. On the side surface of the cavity, temperatures progressively drop away from the bottom surface, but there is no discernible change.
7. The Biot number has little influence on flow and thermal strata since it governs phenomena outside the cavity.
8. Magnetic fields produced the greatest Nu_av_ enhancement on the cavity’s side wall.
9. Increasing the aspect ratio from 1 to 10 reduced Nu by about 23%.
10. The use of upgraded methods consisting of innovative fins and nanoparticles results in an improvement in the melting characteristic.
11. Both the rate of heat transmission and the rate of melting are increased when the inner tube is positioned such that it is at the bottom of the enclosure as well as when the concentration of the nanoparticles is increased.
12. The reduction in PCM’s melting time by roughly 15%, which results from the inclusion of Al_2_O_3_ nanoparticles at a volumetric concentration of 3%, increases PCM melting performance.
13. The optimal area to improve heat transmission is 70% of the hot plate height or 30% of the cold plate height.
14. The double rotations accelerated heat capacity, nanofluid flow, and concentration inside an annulus and altered temperature.
15. Transverse vibrations effectively control heat convection in spinning systems.

## 7. Future Directions

Radiation and free convection dominate the cavity when the external wind is nonexistent or weak. Suppressing heat loss improves cavity and system thermal efficiency. Before this, it was vital to understand radiation and free convection heat transmission. Thus, they were extensively studied. Most studies place cavities sideways or downward. This did not meet the ever-growing application areas of upward-facing cavities, such as second-stage concentrators and lens systems in material surface modifications, solar thermoelectric production systems, solar beam-down tower systems, and testing material behaviors. Upward-facing chambers may cool electronic equipment. There are a great number of pending and important cases that might be mentioned here for the following reasons:

1. There is a possibility that the heat fluxes measured in tests will not match those measured in actual applications, particularly with highly concentrated solar technologies. In the near future, numerical studies with large fluxes will be carried out in order to make up for the current deficit.
2. In subsequent research, it will be possible to determine the effect of the height and width of the flow modulator, the temperature continuity at the blade surface in order to incorporate a two-way thermal coupling, and the variation of the thermo–physical and geometric configurations of the solid domain.
3. Because the movement of nanoparticles alters the local concentration of nanoparticles, an examination of the modification of the local characteristics of the nanofluid that is caused by the movement of nanoparticles may be addressed in future research if it is desired.
4. It is possible that the melting process entails turbulent natural convection in future research, which would correspond to the laminar state that was found in the cavity in earlier investigations.
5. Studies on the entropy and economy of solar cavity receivers may be provided in further works.

## Figures and Tables

**Table 1:** Summary of review on circular cavities.

Authors (Year)	Configuration	Studied Parameters	Study Type	Findings/Highlighted Results
Hadavand et al. (2019) [16]	A new semi-circular lid-driven cavity.	Attack angle, the volume fraction of nanoparticle, and Richardson number.	Numerical (FVM)	The major factor of density difference at Ri = 10 is the cold surface contact with the fluid.
Gangawane and Oztop (2020) [17]	Lid-driven semi-circular cavity with an adiabatic block with different geometries at the center.	The impact of the shape of the adiabatic block, i.e., circular, square, and triangular, on heat transmission.	Numerical (ANSYS)	The block’s presence reduced the cavity’s convection heat transmission.
Dogonchi et al. (2020) [18]	The cavity of semi-circular geometry is filled with ferronanofluid (Fe_3_O_4_-H_2_O).	The effect of nanoparticle volume fraction, heat source/sink parameters, Hartmann number, shape factor, thermal relaxation parameter, and Rayleigh number on fluid flow properties and heat transfer.	Numerical (CVFEM)	The magnetic field may be used to regulate the system.
Hatami et al. (2016) [19]	Circular-wavy cavity	Effects of Nr, Ra, and Le on the isotherm, streamlines, and Nusselt number.	Numerical (FEM)	The suggested FEM with RSM should be useful for optimizing heat transport in irregular geometries.
Sheikholeslami et al. (2018) [20]	Circular cavity with two circular heaters.	Rayleigh number, location angle of heaters, the magnetic strengths ratio, magnetic number, Hartmann number, and nanocomposite particles concentration.	Numerical (FEM)	The host fluid’s Nusselt number increases with MWCNT-Fe_3_O_4_ hybrid nanoparticles.
Ahmadreza et al. (2021) [21]	Newtonian fluid is contained inside a partitioned circular enclosure with a flexible wall.	Rayleigh number and Prandtl number.	Numerical (FEM)	The overall stress applied to the plate is 70 times greater when Pr is increased from 0.71 to 200.

**Table 2:** Summary of review on cylindrical cavities.

Authors (Year)	Configuration	Studied Parameters	Study Type	Findings/Highlighted Results
Bouhal et al. (2018) [22]	A cylindrical cavity incorporates heating sources.	The geometric influence of heating sources and boundary conditions on heat transfer.	Numerical (the physical enthalpy-porosity model)	The fin design increased heat transmission and sped up the melting time of the PCM.
Shen et al. (2017) [23]	Upward-facing cylindrical chambers with isoflux.	Heat flux and tilt angle effects on various aperture diameters.	Experimental and numerical	The changing patterns of radiation heat transfer Nusselt numbers and free convection with heat flux and tilt angle are unaffected by aperture size.
Shen et al. (2015) [24]	Continuous heat flow in an upward-facing cylindrical hollow.	Cavity tilt angle and heat flux.	Experimental, numerical, and empirical	A unique correlation that accurately incorporates the effect of surface heating was initially created to aid many engineering problems when changing surface heating.
Shen et al. (2016) [25]	Continuous heat flow across a cylindrical hollow.	Heat flux and aperture ratio on the tilt angle of the critical cavity.	Experimental and numerical	More characteristics, particularly those within the cavity, should be considered to determine the critical cavity tilt angle.
Vjatkin et al. (2019) [26]	A horizontal spinning cylinder with isothermal boundaries subject to translational vibrations.	Average non-isothermal fluid convection and transverse vibrations in a rotating hollow.	Experimentally and theoretically	Transverse vibrations are effective tools for controlling heat convection in spinning systems.
Xiao et al. (2020) [27]	Cylinder with or without quartz window.	The tilt angles and heated states of the walls.	Experimental	The quartz window helps enhance cavity temperature.
Al-Rashed et al. (2019) [28]	The heated elliptical central cylinder inside a lid-driven hollow.	The solid volume fraction of nanoparticles, a hot elliptical centric cylinder, and cavity angle.	Numerical	The enhancing rate of the Nu from 0 to 45° in base fluid and ϕ = 0.1% and 0.2% is identical to 1.7, 0.8 and 0.3, respectively.
Daabo et al. (2019) [29]	Infrared thermal receiver for small-scale solar Brayton cycle.	Optimize optical and thermal performance.	Numerical (ANSYS) and experimental	A 23% reduction in the heat transfer coefficient of the examined geometry.
Shen et al. (2016) [30]	Isothermal upward-facing cylindrical cavity.	Wind speed, surface temperature, wind incident angle, and cavity tilt angle.	Numerical	The optimal wind incidence angle for maximum loss of combined convective heat depends on the tilt angle of the cavity and wind speed.
Alipourtarzanagh et al. (2020) [31]	A receiver with a heated cylindrical chamber and a set tilt angle.	The effectiveness of an air curtain.	Experimental	An air curtain facing the wind rather than the aperture plane should be used for tilted tower cavity receivers.
Zhang et al. (2022) [32]	An innovative fin structure and Al_2_O_3_ nanoparticles were used in tubular energy storage devices.	Fin layouts and volume fractions of nanoparticles.	Experimental	The use of upgraded methods consisting of innovative fins and nanoparticles results in an improvement in the melting characteristic.
Bouzennada et al. (2021) [33]	The heat transfer fluid may be circulated thanks to the cylindrical enclosure’s inner tube, which is fitted with the necessary hardware.	Inner tube location and nanoparticle concentrations.	Numerical	Both the rate of heat transmission and the rate of melting are increased when the inner tube is positioned such that it is at the bottom of the enclosure, as well as when the concentration of the nanoparticles increased.
Han et al. (2022) [34]	In an annular tube, nanoparticles (Al_2_O_3_-, copper-, and graphene-based nanofluids) are used.	% vol. of Al_2_O_3_-, copper- and graphene.	Experimental	The reduction in PCM’s melting time by roughly 15 percent that results from the inclusion of Al_2_O_3_ nanoparticles at a volumetric concentration of 3 percent increases PCM melting performance.

**Table 3:** Summary of review on hexagonal cavities.

Authors (Year)	Configuration	Studied Parameters	Study Type	Findings/Highlighted Results
Ahmed and Raizah (2021) [35]	Polygonal/polygonal or polygonal/cylinder forms that are inclined.	Entropy, area of flow, and fluid friction.	Galerkin finite element method (FEM)	The increased ratios of entropy tool values due to the variations of *γ* take their high values at minimum values of Ha and *γ* = 30.
Ikram et al. (2021) [36]	A hexagonal air-filled chamber with a constant heat flux floor heater rotated by an adiabatic flow modulator.	An adiabatic flow modulator’s rotation.	Numerical (FVM)	The system response fundamental frequency matches the blade frequency.
Rehman et al. (2020) [37]	The evenly heated T-shaped fin is implanted at the bottom wall of the hexagonal hollow.	The velocity and temperature distribution around uniformly heated T-shaped with Rayleigh number.	Numerical (FEM)	The dimensionless Casson fluid vertical velocity parameter in a vertical T-shaped fin placed in a hexagonal cage boosts Casson fluid.
Haq et al. (2020) [38]	Hexagonal lid-driven hollow with a heated top wall.	Isotherms behavior, the heat transfer rate, and steam lines.	Numerical (FEM)	The Reynold number, heat duration, and Richardson number are linked to heat flow.
Ghalambaz et al. (2019) [39]	The non-uniform magnetic field in a hexagonal container.	Lorentz force, magnetic number.	Numerical (FVM)	Less heat and mass transport when Lorentz force (Hartmann number) increases.
Alia et al. (2017) [40]	The inclined walls of a hexagonal hole are evenly heated while the horizontal walls remain constant.	Average Nusselt number, average temperature, Richardson number, and Hartmann number.	Numerical (FEM)	The average temperature inside the fluid domain and the average Nusselt number at the heated wall vary considerably for the ranged parameter.
Aly et al. (2021) [41]	An annulus is formed by an outside hexagon and an interior, dual curve of various lengths.	The Rayleigh number, Darcy parameter and Hartmann number.	Numerical (ISPH)	Ra increases heat capacity, temperature, and concentration, and Cr curves increase over an annulus.
Aly et al. (2021) [42]	Inner wavy form, outside hexagonal hollow.	The double rotations and magnetic field amongst an outer hexagonal-shaped cavity and wavy inner shape.	Numerical (ISPH)	The double rotations accelerated heat capacity, nanofluid flow, concentration inside an annulus, and altered temperature.

**Table 4:** Summary of review on rectangular cavities.

Authors (Year)	Configuration	Studied Parameters	Study Type	Findings/Highlighted Results
Giwa et al. (2020) [43]	Magnetic induction in a rectangular cavity.	Heat transfer rate, average Nusselt no., heat transfer coefficient, and Rayleigh no.	Experimental	The heat transfer performance depended on strength and direction, φ, AHF deployment, ΔT, and magnetic induction.
Elsherbiny and Ismail (2015) [44]	Two localized heat sources in inclined rectangular chambers.	Effect of tilt angle and aspect ratio on laminar natural convection.	Numerical	The cavity may be tilted 60° from horizontal to maximize Nu.
Soares et al. (2015) [45]	A vertical stack of rectangular cavities.	The period of thermal regulation and control-temperature value on the hot surface.	Experimental	Subcooling is critical for solidifying the free PCM during the discharging process and must be considered while modeling.
Giwa et al. (2020) [46]	Magnetic excitation of a rectangular cavity.	The thermo-convection heat transfer behavior.	Experimental	The findings are strongly related to ΔT, φ, magnetic excitation strength, use of HMNFs, the configuration of magnetic excitation, and position.
Li and Tong (2016) [47]	Low width-to-height ratios in inclined rectangular cavities.	Cavity inclination and width/ height ratio.	Experimental and CFD	The effect on natural convection flow is minimal when the width-to-height ratio of the cavity surpasses 4.
Qi et al. (2020) [48]	rectangular hollow with thermally insulated walls on one side and heat transfer fluid flowing on the other.	Stefan number or Reynolds number.	Numerical	A series of dimensionless correlations depict the changing modified Nusselt number and law of melting fraction against time.
Li et al. (2020) [49]	NePCM in rectangular cavities that are differentially heated.	The cavity has two height-to-width aspect ratios.	Numerical	As the cavity becomes narrower, the advantage of increased heat conduction may be more completely used, lowering the contribution of natural convection.
Thiers et al. (2020) [50]	Rectangular differentially heated cavity.	Influences in the vertical location of the disturbance area and wave characteristics.	Numerical	The optimal area to improve heat transmission is 70% of the hot plate height or 30% of the cold plate height.
Li et al. (2021) [51]	There is a continuous heat flux on one side, while on the other side, there is a flow boundary condition.	The influences of the aspect ratio, Rayleigh number, and Reynolds number.	Numerical	The amount of cold and hot fluids combined in the cavity influence local heat transfer at the wall.
Qin et al. (2021) [52]	Heat transfer fluids flow from the bottom up in a rectangular chamber.	The melting process of phase change material.	Numerical	Reynolds and Rayleigh numbers play the main role in the melting of PCM.

## Data Availability

The data used to support the finding of this study are included within the article.

## Abbreviation

**Symbol**	**Definition**
AR	Aspect ratio
Bi	Biot number
CFD	Computational fluid dynamics
CVFEM	The control volume finite element method
FEM	Finite element method
FHD	Ferro-hydrodynamic
Ha	Hartmann number
HTF	Heat transfer fluid
MHD	Magnetohydrodynamic
Mn_f_	Magnetic number
MWCNT	Multi-wall carbon nanotubes
NePCM	Nano-enhanced phase change material
Nu	Nusselt number
PCM	Phase change material
Pr	Prandtl number
Ra	Rayleigh number
Ri	Richardson number
α	Angle of cavity
γr	Magnetic strengths ratio parameter
φ	Tilt angle
ϕ	The volume fraction of solid nanoparticles
